# CAT-Site: Predicting Protein Binding Sites Using a Convolutional Neural Network

**DOI:** 10.3390/pharmaceutics15010119

**Published:** 2022-12-29

**Authors:** Žan Hafner Petrovski, Barbara Hribar-Lee, Zoran Bosnić

**Affiliations:** 1 University of Ljubljana, Faculty of Computer and Information Science, SI-1000 Ljubljana, Slovenia; 2 University of Ljubljana, Faculty of Chemistry and Chemical Technology, SI-1000 Ljubljana, Slovenia

**Keywords:** protein binding site prediction, ligands, molecular docking, machine learning, convolutional neural network

## Abstract

Identifying binding sites on the protein surface is an important part of computer-assisted drug design processes. Reliable prediction of binding sites not only assists with docking algorithms, but it can also explain the possible side-effects of a potential drug as well as its efficiency. In this work, we propose a novel workflow for predicting possible binding sites of a ligand on a protein surface. We use proteins from the PDBbind and sc-PDB databases, from which we combine available ligand information for similar proteins using all the possible ligands rather than only a special sub-selection to generalize the work of existing research. After performing protein clustering and merging of ligands of similar proteins, we use a three-dimensional convolutional neural network that takes into account the spatial structure of a protein. Lastly, we combine ligandability predictions for points on protein surfaces into joint binding sites. Analysis of our model’s performance shows that its achieved sensitivity is 0.829, specificity is 0.98, and $F_{1}$ score is 0.517, and that for 54% of larger and pharmacologically relevant binding sites, the distance between their real and predicted centers amounts to less than 4 Å.

## 1. Introduction

The rapid development of targeted drugs and immunotherapies in the last years presents significant progress in treating different diseases. However, the cost of these drugs is still extremely high, which hinders their accessibility to more patients on an everyday basis [1]. One element that contributes significantly to this high cost is the long and expensive process of drug development and the high attrition rate of new chemical entities due to poor validation of drug targets [2]. To save time and money with drug discovery and development, more and more drug discovery processes today begin with computation rather than test-tube experimentation: so-called virtual screening of potential drug candidates is performed through molecular docking studies. Using molecular docking for predicting the preferred orientation of a designed molecule (ligand) to a receptor can save time and money and is therefore widely used in the field of pharmacology [2,3,4]. Even though the designing of drug molecules is done in such a way to alter the activity of the specific target (specific binding site on the protein molecule), the drug molecule, when introduced into the body, interacts and also binds with other parts of the target protein molecule as well as to other proteins, which can lead to reduced efficiency or even severe side effects of the particular drug [5]. On the other hand, in certain cases, this so-called off-target binding has been found to have multiple beneficent effects as well [6]. Well-designed docking studies that predict all possible biding sites on the target protein molecule can therefore foretell the side effects as well as other uses of designed drugs.

Two aspects are crucial for efficient docking programs: search algorithms and scoring functions. The search algorithms analyze and generate different orientations (poses) of ligands and binding site locations on a target protein. The scoring functions are then used to rank the ligand–receptor pairs [7]. While in recent years much effort has been put into improving the scoring functions as well as pose-generating algorithms, the search algorithms for identifying the binding sites on the protein molecules are often overlooked [5]. The most common way to address this problem is through computer simulation of molecular dynamics, which is generally used for so-called blind-docking computer experiments. The method, however, is extremely time-consuming, and the results are often strongly dependent on the force-fields used [8]. Therefore, in the last couple of years, machine-learning methods as an alternative have been more and more frequently implemented to optimize the docking process [9]. However, despite their success, they are usually limited in use for only relatively small populations of well-defined ligands [10]. In this work, we propose a more general approach to tackle this problem.

### 1.1. Related Work

Predicting protein binding sites has been an active field of research for a few decades in bioinformatics, and researchers have come up with different ways to solve the problem. The authors of FPocket [11] use a geometric method to predict potential binding sites. Their main idea is the use of α-spheres, which are defined as three-dimensional spheres with four atoms on their surface and none in the interior. By a method of clustering, they group them into potential binding sites. A different approach is used for P2RANK [12], where the authors assign an attribute vector to each point and then compute 34 properties for the surface points, considering the adjacent atoms, their bonds and biochemical properties. The approach uses a random forest algorithm with 100 trees of unrestricted depth and 6 attributes. The predicted ligandable points are afterwards clustered, and the clusters are ordered according to the properties of the containing points. The work states that P2RANK performs better than its predecessor PRANK [13] and FPocket [11].

Another approach, DeepSite [14], uses a convolutional neural network (CNN) that takes as an input the three-dimensional representation of the protein. Even though the first steps in this method (i.e., determining the collection of points and then predicting their ligandable properties) are the same as in P2RANK, the points in DeepSite do not lie only on the protein but are distributed uniformly within the bounding box. A point is classified as a ligandable site if it is within 4Å from the geometric center of the ligand. The convolution network consists of the sequence of convolutional, pooling, dropout, and fully connected layers. The input is comprised of eight property channels of size 16 × 16 × 16, while the output has a value between 0 and 1, with a larger value meaning more certain prediction of ligandability. Each channel represents a different molecular property in the vicinity of a particular point. The authors show that the method gives better results than FPocket.

In DeepPocket [15], the geometric method of FPocket is combined with the machine learning approach of a convolutional network to predict the ligandability in three steps. In the first step, Fpocket is used to generate potentially ligandable points, which are ranked by their quality using the first convolutional network. The output is a value between 0 and 1, with a larger value meaning more certain prediction of ligandability. The network architecture used for this prediction is relatively simple, and the voxelization of the input is similar to DeepSite except that the library Libmolgrid [16] is used and more biochemical molecular properties are included through more channels. The best-ranked ligandable sites are used as an input into the second segmentational neural network, which determines whether the input points are ligandable or not. The architecture of the second neural network relies on the U-Net [17] network idea that includes the connection between non-consecutive layers.

A different approach is used in the AK-Score program, where the strength of the interaction between protein and ligand in a certain mutual position is predicted. This information can then be used in practice for designing a ligand that would bind to the protein as strongly as possible. The input is similar to that of DeepSite and DeepPocket; the output, however, is the numerical value corresponding to the binding strength. As such, the method solves a regressional problem. The results are evaluated using Grad-CAM [18], which employs the output activation of the last convolutional layer and the gradient of the final prediction. The method allows the assessing of different ligand–protein interface contributions to the predicted values.

### 1.2. Aim and Contributions of This Work

In our work, we merged the idea of viewing points on the protein surface as done in P2RANK and the use of a CNN that takes a voxelized protein as its input from DeepSite. The use of surface points allows the CNN to specialize more to such cases. We diverge from the aforementioned works in how we choose and manipulate our dataset. We choose more proteins and use all their known ligands, not only the selected, usually larger ones. Additionally, we increase the average number of ligands per protein by finding the set of ligands observed on similar proteins and then use the whole set on one single protein. In this way, we decrease the chance of an actual binding site being marked as a nonbinding site, i.e., a false non-ligandable location on a protein’s surface. We also cluster proteins with a lower similarity threshold to prevent data leakage between training and test datasets, which makes our results more robust. To model ligandability points on a protein, we use a 3D convolutional neural network, which is one of the well-established models in machine learning, especially in the field of computer vision. Our model is trained for the first time on the new and original dataset, which allows it to capture new knowledge and insights that are different from the results of the current state-of-the-art models.

Another important step is the final clustering of surface points by prediction of their ligandability. Our algorithm is based on statistical analysis of binding sites and uses adaptable thresholds to decide whether a point is part of a binding site or not. We name the entire method CAT-Site, as it consists of ***c**leaning* the dataset, ***a**ligning* proteins and ligands, ***t**raining* of the model, and finally predicting the binding *site*. In this work, we therefore reassess the current state-of-the-art for protein binding site detection and propose a novel method of protein clustering and merging of ligands of similar proteins. We aim to apply several insights about the drawbacks of the existing methods to evaluate our machine learning model in a more robust manner.

The work in this paper presents the following main contributions:

- A data preprocessing method and novel method for alignment of aminoacid sequences and their clustering;
- Representation of molecules for machine learning and modeling them with a CNN that receives the three-dimensional images with eight channels as its input;
- The novel method for final combining of ligandability predictions into binding sites by processing the output of the CNN.

## 2. Materials and Methods

**Figure 1:**
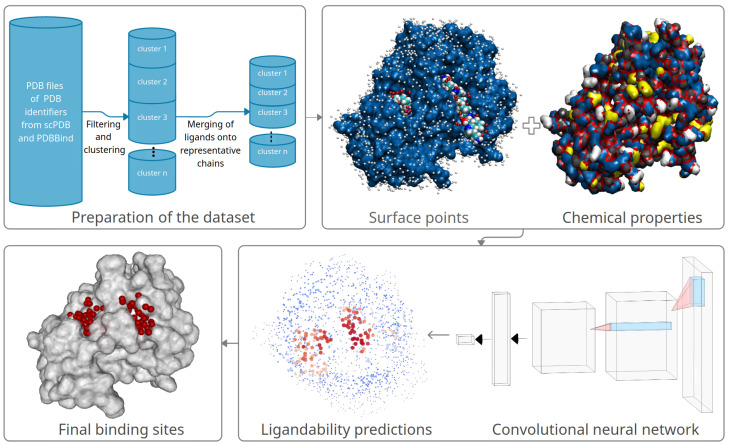
A high-level overview of the proposed method: it consists of preparation of the dataset, followed by surface points and chemical properties computation, training a convolutional neural network, predicting ligandability probabilities of surface points, and clustering the predicted ligandable points into predicted binding sites.

In this section, we describe in detail how we prepare our data and use it for training the convolutional neural network (CNN). First, we clean the training data and align and combine the contained proteins and ligands. Next, we model the ligandability using the CNN. In the last step, we combine ligandability predictions into predicted binding sites. Figure 1 illustrates the entire method’s workflow, which we describe in detail in the following subsections.

### 2.1. Selection and Preparation of Training Data

To create our dataset, we used the PDB identifiers that are present in the PDBBind [19] and the scPDB [20] databases. However, since only one binding site was occupied by a ligand in these databases, we downloaded the full unprocessed data for the selected PDB identifiers from the extended RCSB PDB [21] database, which allowed us to collect information about all possible binding sites. As we know, proteins often consist of more than one chain. It is not unusual that some of the chains are identical in their structure and consequently have their binding sites at the same locations. For this reason, it would be impractical to predict binding sites on the whole proteins; instead, we look at each chain separately.

In the preliminary dataset analysis, we noticed that some chains were not appropriate for use: some chains were too small, and some were attributed ligands that are too far from the chain to have any interaction. To solve these issues, we filtered our dataset by choosing chains with at least 28 aminoacids and ligands with at least 7 non-hydrogen atoms. Another condition for ligands was that the minimum distance between ligands’ atoms and chains’ C${}_{\alpha }$ atoms was at most 5 Å. With these conditions, we also managed to include the small insulin molecule on the hand, and remove water molecules, ions, and other small particles that often float around proteins (e.g., glycerol with 6 atoms) on the other hand. After filtering, we were left with suitably large chains with ligands that were very likely to have an actual interaction with the chain. If we chose to include smaller ligands such as water and ions present only for the process of determining the molecular structure, we would predict non-ligandable locations as ligandable.

An even more difficult problem is preventing the occurrence of false non-ligandable locations. Since binding sites of a chain are only rarely all occupied, we cannot mark the non-occupied locations as non-ligandable with certainty. To mitigate this problem, we narrow the set of chains by merging ligands belonging to chains with enough similarity by clustering them based on their amino acid sequences. On each of these clusters, we afterwards merged the ligands. Note that the clusters computed at this step were also used at splitting the dataset into training, validation, and test sets.

### 2.2. Alignment of Amino Acid Sequences and Their Clustering

Aligning amino acid sequences is a broad topic that studies the structural similarity among proteins. To cluster protein chains, we used the MMseqs2 [22] library, which offers sufficient control throughout the process. Since our dataset is relatively small compared to the datasets this library can be used on, we were able to skip the prefiltering step and ran the Smith–Waterman algorithm [23] on all protein pairs. In short, for a pair of aminoacid sequences

$$\begin{array}{cc} A & =a_{1}a_{2}\ldots a_{n},\\ B & =b_{1}b_{2}\ldots b_{m}, \end{array}$$

where *m* and *n* are the lengths of the initial sequences, the algorithm creates a matrix in which the values represent the highest similarity—according to the selected substitution matrix (e.g., BLOSUM62 matrix)—between different subsequences of *A* and subsequences of *B*. The alignment of two subsequences is optimized by taking into account the matches or mismatches, insertions, and deletions of the amino acids. The Smith–Waterman algorithm follows the dynamic programming paradigm to compute the values of the matrix. To find the pair of the aligned sequences $A^{\prime }=a_{1}^{\prime }a_{2}^{\prime }\ldots a_{N}^{\prime }$ and $B^{\prime }=b_{1}^{\prime }b_{2}^{\prime }\ldots b_{N}^{\prime }$, where *N* is the length of the aligned sequences, it finds the highest value in the matrix and then follows the path via which it came to this value during the computation process. The details of the algorithm are explained in the original paper [23].

The similarity of initial sequences is then defined as

$$S\left(A,B\right)=\frac{\left|\{k\;|\;a_{k}^{\prime }=b_{k}^{\prime },k\in \left\{1,2,\ldots ,N\right\}\}\right|}{N}.$$

To avoid cases where similarity measure is high but the actual aligned sequences cover only a small part of the initial sequences, we define the covering *c*. For the initial sequence *A* and aligned sequence $A^{\prime }$ where $A^{\prime }$ starts on index $n_{1}$ of *A* and ends on index $n_{2}$, the covering $c(A,A^{\prime })$ is defined as

$$c\left(A,A^{\prime }\right)=\frac{n_{2}-n_{1}+1}{n}.$$

Now let $\bar{s}$ denote the lower bound of similarity and $\bar{c}$ the lower bound of covering. For each pair of sequences $\left(A_{i},A_{j}\right)$, i,j∈{1,2,…,z}, from the set of sequences $Z=\{A_{1},A_{2},\ldots ,A_{z}\}$, we compute the similarity $S(A_{i},A_{j})$ and coverings $c(A_{i},A_{i}^{\prime })$ and $c(A_{j},A_{j}^{\prime })$, where $A_{i}^{\prime }$ and $A_{j}^{\prime }$ are corresponding aligned sequences.

Let G=(V,E) be an undirected graph, where the set of vertices *V* is the set of sequences *Z* and the set of edges *E* where edge $\left(A_{i},A_{j}\right)$ is in *E* if the following conditions hold:

(1) $$\begin{array}{cc} S(A_{i},A_{j}) & >\bar{s},\\ c(A_{i},A_{i}^{\prime }) & >\bar{c},\\ c(A_{j},A_{j}^{\prime }) & >\bar{c}. \end{array}$$

Since our goal is to cluster similar sequences, we define as clusters the connected components of *G*. The sequences *A* and $A^{\prime }$ are therefore in the same cluster if there exists a path between them. This way of clustering ensures that each pair of similar sequences is in the same cluster. Stated differently, for two different clusters $G_{1}$ and $G_{2}$, sequences $A_{1}\in G_{1}$ and $A_{2}\in G_{2}$ for which the conditions in Equation (1) hold do not exist. In our case, we set the lower bound of similarity to $\bar{s}=0.8$ and the lower bound for covering to $\bar{c}=0.9$. The two parameters correspond to min-seq-id and c in the MMseqs2 library.

We believe that sequence alignment with the Smith–Waterman algorithm from the MMSeqs2 library returned very robust results. Thus, we do not expect some other algorithm to give significantly different or better results. One could experiment with different amino acid substitution matrices (e.g., different types of BLOSUM matrices), but for our preliminary experiments, the outcome should not vary significantly. One could also consider the depth of a residue as a parameter by which to group residues at the evaluation phase (for example, one group would consist of residues lying close to the visible surface and the other of residues that are buried).

### 2.3. Merging of Ligands of Similar Chains

**Figure 2:**
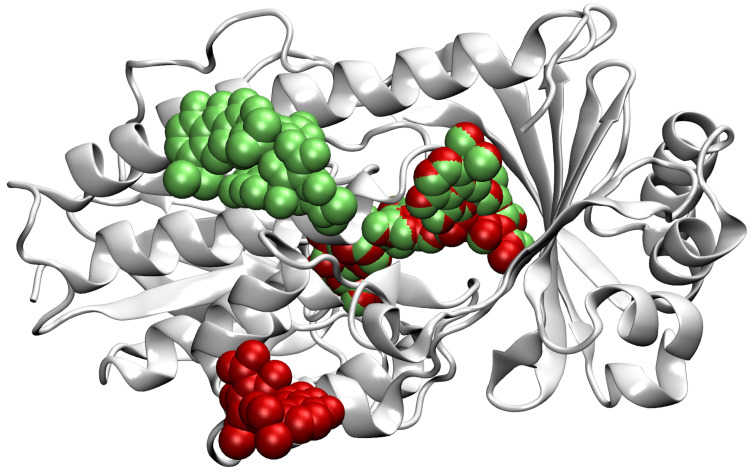
A representation of merging of ligands to the chain *D* of the PBD file 4a99 [24,25]. Ligands originally on the chain *D* are shown in green and ligands from the chain *A* are red. We see that the two chains have the right ligand in common and that the other two ligands originally had only one of them present. In this specific case, we kept the chain *D* with its original two ligands and added the lower ligand in red. For the representation, we use the VMD [26] software.

In our dataset, there are proteins that are represented in more than one PDB file but not necessarily with the same set of ligands, or ligands bound to the same binding sites. Cases in which a protein consists of multiple identical copies of some chain are also present. In order to increase the informative value of each chain, we merge the ligands of similar chains onto one representative chain. This way, we reduce the number of locations falsely marked as non-ligandable, increase the number of taken binding sites on each kept chain, and remove redundant data. Note that the PDF files do not allow certain deducing of non-ligandable locations; conversely, we treat only locations with present ligands in any input file to be certainly ligandable. If for some chain *A* there exist different proteins with different numbers of identical copies, we can represent all their binding sites with only the representative chain *A*, on which we add ligands that were originally bound to other chain instances. Since the chain *A* carries information of all its identical copies, these copies can be removed from the dataset. Through this process, we achieved higher information density and decreased the occurrence of highly similar data entries. An example of the merging of ligands of two identical chains is shown in Figure 2.

**Figure 3:**
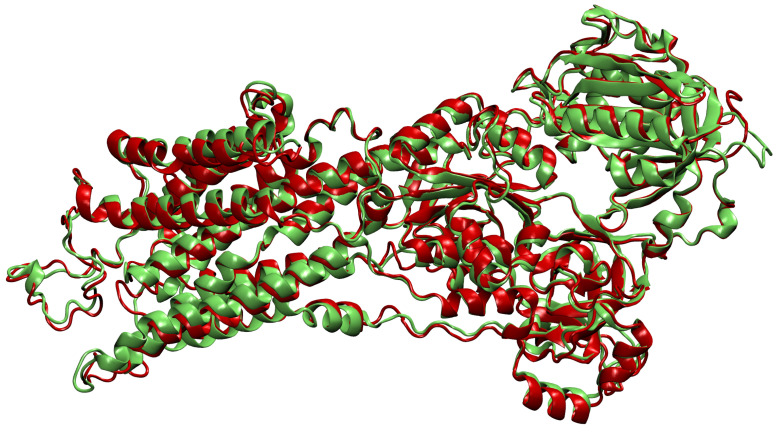
Oriented chains *A* from PDB files 2eat [28,29] (red) and 2eau [28,30] (green). The amino acid sequences of these two chains are identical. We see that in some areas the chains overlap very closely, but some deviation is allowed as well—for example, in the upper left helix. If we used a higher threshold for the rmsd distance, more deviation would be possible and, consequently, ligands would possibly be merged onto wrong locations. For visualization, we used the VMD software [26].

Our merging algorithm takes as its input a set of PDB files. We run it independently on each cluster of resulting chains from Section 2.2. The algorithm has the following main steps:

1. We read and sort the chains from the cluster in descending order based on the number of bound ligands and the total number of atoms of bound ligands. Sorting by these two criteria, we prioritize chains with more bound ligands, and in case two chains have the same number of ligands, we prioritize the chain with larger ligands on average. The reason for the second sorting criterion is that ligands bound to approximately the same binding site can be significantly different in size. If in such a case we chose the smaller ligand as the representative occupant of the binding site, a large part of the actual binding site would be falsely marked as non-ligandable.
2. We choose the representative chain based on the number of chains from the cluster to which it can be aligned well. The basic idea is to find a chain from the cluster that can be aligned to a sufficient number of other chains in its cluster. We follow the order in the sorted list of chains described in the previous step until we find a chain that fits our criteria. The search for the common orientation of chains is done by adapting the method prody_align from the ProDy library [27]. This method receives as an input a reference chain and a list of chains that we wish to rotate and translate so they align with the reference chain. The algorithm also takes the lower bound for sequence similarity seqid=0.9 and the lower bound for covering overlap=0.9, which correspond to the parameters $\bar{s}$ and $\bar{c}$ from Section 2.2.
3. Then, we add ligands to the selected reference chain from sufficiently similar chains. We only add ligands that do not intersect with the ligands already bound to the reference chain. We also discard ligands that intersect with the reference chain or are too far away. In this part, we consider two chains as sufficiently similar if the root mean square deviation of pairs of the matching C${}_{\alpha }$ atoms is smaller than 2 Å. That is,
  (2) $$\mathrm{rmsd}\left(p_{1},p_{2}\right)=\sqrt{\frac{\sum_{i=1}^{n}d_{2}{\left(p_{1}\left[i\right],p_{2}\left[i\right]\right)}^{2}}{n}}<2,$$
  where $p_{1}$ and $p_{2}$ are lists of matching C${}_{\alpha }$ atoms, $n=|p_{1}\left|=\right|p_{2}|$, and $d_{2}$ is the euclidean distance between two points. The upper bound of 2 was set empirically, taking into account the alignment or oriented pairs of chains. An example where $\mathrm{rmsd}(p_{1},p_{2})=1.8$ Å is shown in Figure 3. The chains align sufficiently well despite the value being close to the upper bound.
4. If we were not able to align all chains in the cluster to a single reference chain, we repeat the process on the remaining chains—we assign the remaining chains to the new cluster and go back to the first step.

The described procedure resulted in 6169 chains clustered into 5284 clusters.

### 2.4. Representation of Molecules for Machine Learning

For presenting the resulting protein/ligand data to a machine learning algorithm, we used a spatial description. This means that each protein is described by a three-dimensional image with a number of channels, where each *channel* represents a property in the vicinity of a certain point. If we look at a protein as a set of points in space, we can surround it with a bounding box with some padding on its sides. In this bounding box, we then choose equidistant points on a rectangular grid and compute chemical properties at their locations. This is done by an algorithm from the HTMD library [31] that takes into account the atoms’ locations, their elements, bonds between them, and other properties. A condensed description of different atom types and their grouping based on chemical properties is shown in Table 1 and Table 2, respectively.

**Figure 4:**
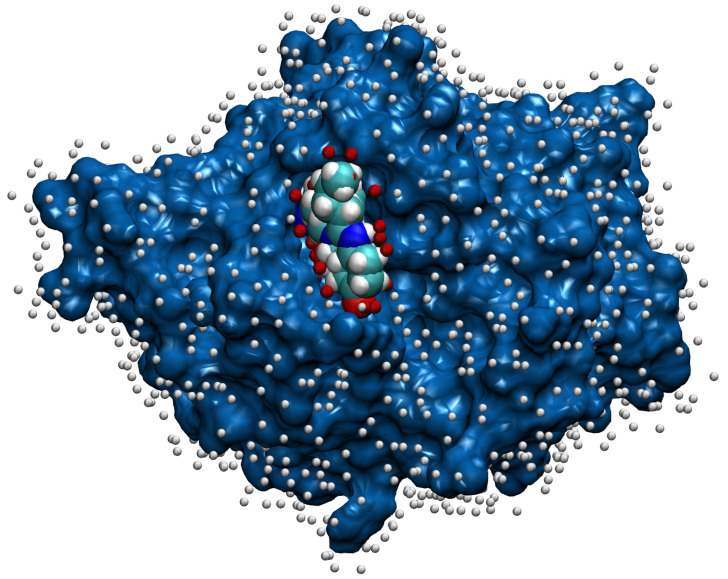
The surface of chain *A* for the PDB file 1a8t [34,35] is painted blue, and the bound ligand that represents its elements has default colors. The white balls close to the surface denote non-ligandable points, and the red balls denote ligandable points. For the representation, we used the VMD [26] software.

Along with computation of the proteins’ attributes, we also need to mark each chain’s true ligandable locations. We do that by reading only ligands from the original PDB file that were left after initial pre-processing and filtering. The binding of the ligands takes place on the surface of the protein molecule, which is usually described as the protein solvent-accessible surface (SAS) [32]. The points on the SAS of the protein were calculated using the NumericalSurface method from the open-source library CDK [33], which takes the solvent radius and tessellation level as input parameters. Having computed the surface points, we chose to define a point on the chain’s surface ligandable if the excluded volume property had a value higher than 0.0001 for some of the chain’s ligands. While this threshold was set empirically, it does also have a meaning—for an atom of a ligand, it is reached at the distance from the atom’s center, which is about twice the size of the atom’s radius. An example of the surface points together with their ligandability property is shown in Figure 4.

**Figure 5:**
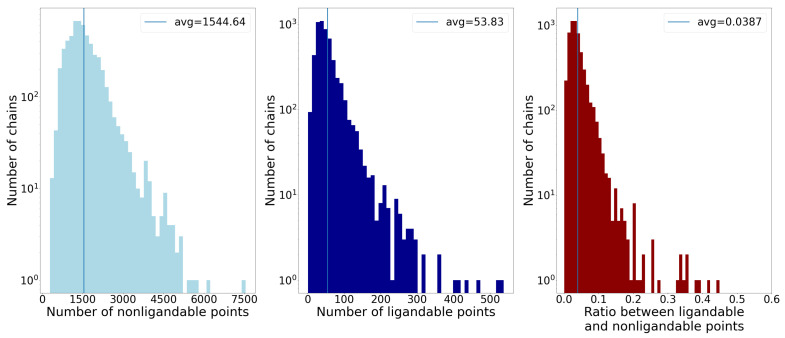
The first two histograms show the number of non-ligandable and ligandable points by the number of chains, and the third one shows the ratio between ligandable and non-ligandable points. The average of each property is represented by vertical lines. The main takeaway is that on average less than 4% of the points are ligandable, which presents a problem when sampling data for machine learning.

To gain some intuition about the processed set of chains and their surface points, Figure 5 shows histograms of their basic properties. Usually, only a small part of the surface allows ligands to bind; consequently, there are more non-ligandable than ligandable points.

### 2.5. Machine Learning with Convolutional Neural Network

The convolutional neural network (CNN) as a machine learning model is able to receive our pre-processed dataset of three-dimensional images with eight channels as its input. Each channel represents a chemical property derived from atom types set by the openbabel [36] library. A short description of different atom types and their grouping based on chemical properties is shown in Table 1 and Table 2, respectively. In our work, we used the PyTorch library [37] for the implementation of the CNN. Our model consists of four convolutional and two pooling layers for feature extraction and two dense layers for classification. On the output of each convolutional and dense layer, we applied the ReLU activation function and batch normalization; a dropout layer was also used. A single input to the CNN is described by eight cubes—one for each channel representing the chemical properties of the protein—of shape 19×19×19. These cubes are centered at a selected point on the accessible surface area of the protein, which gives the model the information needed to assess the surroundings of the selected point. As the output, the CNN gives the classification into *ligandable* and *non-ligandable* classes (as a vector with two values that sum up to 1).

Note that we considered different CNN architectures for our work, but in the preliminary testing the alternatives revealed worse performance. The final version is mainly characterized by having a large number of channels and not so many layers—increasing the number of channels turned out to be the most effective way to increase the quality of predictions, while increasing the number of layers heavily affected the running time of the training process. We also compared the different pooling types, where max-pooling performed better than average-pooling.

For hyperparameter search and evaluation, we used the nested cross-validation technique. We split our dataset ten times into three disjoint parts—the training, validation, and test set. Each training set consisted of 80% of all clusters of chains, while the validation and test set each contained 10%. We made sure that all of the ten test sets were pairwise disjoint. We therefore considered each triple of test, validation, and training sets to be independent in the process of nested cross-validation, since the clustering method described in Section 2.2 minimizes the chance of data-leakage between different clusters. Pairs of training and validation sets were used in the process of hyperparameter optimization; the best set of parameters was then used to train the model on the union of the training and validation set and was then evaluated on the test set. The optimal hyperparameters were chosen based on the average value of the loss function of the validation set in the last five epochs of training—the total number of epochs was set to 40. Even though the training sets consisted of almost 5000 clusters, we only used 1024 in each epoch because of the time-consuming training process. Training of a single model took from 30 min up to two hours depending on the choice of hyperparameters.

When training a machine learning model, it is important to pay attention to the learning rate. We chose to follow the super-convergence method [38], where at the first few iterations the learning rate is slow but rises gradually to the maximum value, and in the last phase it descends to a small value to stabilize the learning process. When choosing samples for the batch in each iteration, we first randomly picked $n_{c}$ clusters from the training set, one chain from each cluster, then from each of the selected chains, $n_{p}$ points on the chain’s surface. The total number of samples—cubes centered at the points on the surface—in a batch is therefore $n_{c}\cdot n_{p}$. The pairs of $n_{c}$ and $n_{p}$ that we tested for optimality, namely (16,32), (32,16), (64,8), and (128,4), all had the same product of 512. So altogether we had three hyperparameters to tune: learning rate, $n_{c}$, and $n_{p}$. For $n_{c}$ and $n_{p}$, the pair (128,4) was never the optimal choice since it caused overfitting to the training set and high deviation of the loss on the validation set because of the relatively low number of weight updates per epoch. The pair (16,32) was chosen twice and the other two pairs four times each.

Additionally, as we saw in Figure 5, our dataset is unbalanced when we look at ligandable and non-ligandable points. This could lead to bias if we simply chose random points from the chains’ surface. For this reason, we chose to oversample the ligandable points so that the number of ligandable and non-ligandable samples fed to the model during training was the same.

### 2.6. Combining Ligandability Predictions into Binding Sites

Our algorithm for combining ligandability predictions into binding sites is based on statistical analysis of binding sites in our dataset. For points on the SAS of each binding site, we analyzed the following properties:

- Number of points;
- Average distance of points from the geometric center;
- Standard deviation of distance of points from the geometric center.

**Figure 6:**
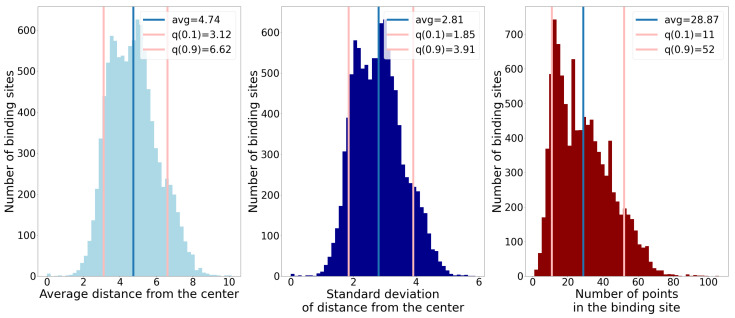
Histograms of three different properties of binding sites. The blue lines denote the average value, and the pink lines denote the 10th and 90th percentiles.

Each of these properties offers some information on the size and shape of binding sites. The standard deviation of distance tells us if a binding site’s shape resembles a sphere (small value) or if it is elongated (large value). In Figure 6, histograms of the described properties are shown. We also denoted the 10th and 90th percentiles to show the chosen boundaries that our predicted binding sites were supposed to suffice. We see that the observed properties do not have peaks too far apart, and that inside the interval between the lower and upper bound the data are dense, which is beneficial for our method.

The pseudocode for the proposed algorithm is given in Algorithm 1. It uses the bounding values for the described properties to combine pointwise predictions into final binding sites. The function binding_sites takes the pointwise predictions $y_{p}$ for points as input. The set of points is split into a ligandable or a non-ligandable class based on the threshold value *c*. In the while loop, points from ligandable class are clustered into components based on the pairwise distance of points. We look at the points as vertices of a graph, and for the edges, we state that two points are connected if the distance between them is smaller than 4 Å. The formed clusters are the connected components of this graph. Then, we check if a cluster fits into the bounds set by percentiles of all three properties; if it does, we add it to the predicted binding sites and remove its points from the points variable.

Based on the condition, we change the variables *a* and *b* that serve as the boundaries of the interval in which the threshold *c* varies. On the remaining set of points, we then compute the new ligandable points based on the updated threshold and compute the new condition for the while loop. Computing the condition is done with the method compute_condition that tells us whether we should break the while loop or change the interval. The decision to change the interval is made on the basis of the percentiles and the shape of the current clusters: increasing *a* leads to increasing *c*, and decreasing *b* leads to decreasing *c*. Intuitively, we can think of this process as extracting the “well shaped” clusters from the current iteration and then setting up the threshold so that more clusters can be extracted in the following iterations.
**Algorithm 1** Grouping predicted ligandable points into binding sites.1: **function** binding_sites($y_{p},points$)
2:     a←0.3           ▹ Real values *a* and *b* define the interval in which we look for the best *c* value.3:     b←1
4:     c←0.5    ▹ Real value *c* is the threshold for classification of ligandability predictions in each iteration of the while loop.5:     ligandablePoints←
select_ligandable_points($y_{p},points,c$)6:     bindingSites←emptyList7:     iteration←08:     condition←“continue”9:     **while** condition is not “break” **do**10:         clusters←
components(ligandablePoints)11:         **for** cluster∈clusters **do**12:              **if** cluster properties fit into quantile bounds **then**13:                  append cluster to bindingSites14:            **end if**15:         **end for**16:         points←points−bindingSites.points17:         **if** condition is “increase *a*” **then**18:              a←0.2·(b−a)+a19:         **else if** condition is “decrease *b*” **then**20:              b←0.2·(a−b)+b21:         **end if**22:         c←(a+b)/223:         iteration←iteration+124:         ligandablePoints←
select_ligandable_points($y_{p},points,c$)25:         condition←
compute_condition(clusters,iteration,#points)26:     **end while**27:     **return** bindingSites28: **end function** 29: **function**
compute_condition(clusters,iteration,#points)30:      **if** iteration>20 **then return** “break”31:      **else if** $\frac{\mathrm{len}(clusters.points)}{\#points}>0.2$ **then return** “increase *a*”32:      **else if** clusters is an emptyList **then return** “decrease *b*”33:      **else if** $\max\left(\mathrm{size}\left(clusters\right)\right)<q_{v}\left(0.1\right)$ **then return** “decrease *b*”34:      **else if** $\max\left(\mathrm{size}\left(clusters\right)\right)>q_{v}\left(0.9\right)$ **then return** “increase *a*”35:      **else if** $\max\left(\mathrm{avg\_distance}\left(clusters\right)\right)<q_{a}\left(0.1\right)$ **then return** “decrease *b*”36:      **else if** $\max\left(\mathrm{avg\_distance}\left(clusters\right)\right)<q_{a}\left(0.9\right)$ **then return** “increase *a*”37:      **else if** $\max\left(\mathrm{std\_distance}\left(clusters\right)\right)<q_{s}\left(0.1\right)$ **then return** “decrease *b*”38:      **else if** $\max\left(\mathrm{std\_distance}\left(clusters\right)\right)<q_{s}\left(0.9\right)$ **then return** “increase *a*”39:      **else**40:           **return** “decrease *b*”41:     **end if**42: **end function** 43: **function** components(points)44:     V←points45:     $E\leftarrow \{\left(x,y\right)\;|\;d_{2}\left(x,y\right)<4\}$            ▹ Two points are connected if they are closer than 4Å46:     G←(V,E)                                                       ▹ Create a graph with vertices *V* and edges *E*47:     **return** connected_components(G)48: **end function** 49: **function** select_ligandable_points($y_{p},points,c$)50:     ligandablePoints←emptyList51:     **for** point∈points **do**52:         **if** $y_{p}\left[point\right]>c$
**then**                  ▹ Check if prediction for point is higher than threshold *c*53:              append point to ligandablePoints54:         **end if**55:     **end for**56:     **return** ligandablePoints57: **end function**

**Figure 7:**
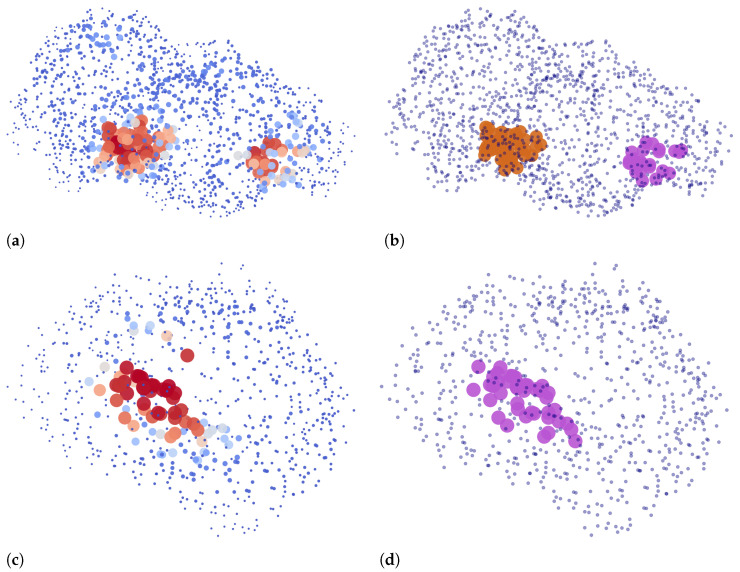
The input and the output of Algorithm 1: Two left figures show points on the protein surface with respect to their predicted ligandability, where higher values are denoted by larger and darker red points. Two corresponding right figures show the same two proteins, denoting the predicted binding sites that are returned by the algorithm based on ligandability predictions. Points that are a part of a binding site are drawn with larger circles. Different binding sites for each chain are colored differently with either purple or brown. Other points are denoted with blue dots. (**a**) Predictions for 3nk7, chain B [39,40]. (**b**) Predicted binding sites for 3nk7, chain B. (**c**) Predictions for 4rrg, chain C [41,42]. (**d**) Predicted binding site for 4rrg, chain C.

Two examples of our binding site extraction algorithm are shown in Figure 7, where we plot point clouds based on ligandability predictions and their clustering into detected binding sites.

## 3. Results

In our work, we performed the evaluation differently than performing a typical evaluation of the classifiers. Due to the nature of the problem, the metrics that are derived from the confusion matrix do not clearly reflect the performance of binding site prediction. To evaluate the binding site prediction performance, we thus applied two more relevant metrics: (1) the distance between the prediction and the actual binding site and (2) the accuracy of the predicted point ligandability. However, since the output of our convolutional neural network is merely ligandability predictions for individual points (and not binding sites), we need to group these predicted points into binding sites, as described in Section 2.6.

### 3.1. Distance from the Center and Discrete Volumetric Overlap

In the field of predicting binding sites, distance between the geometric centers of points of the predicted binding site $V_{p}$ and points of the true binding site $V_{t}$ (DCC—distance center–center) and discrete volumetric overlap (DVO) are the two frequently used metrics [11,12,14,15]. The DVO metric is expressed as a quotient between the number of points in their intersection and the number of points in their union. In this way, the former metric expresses how well the general location of clusters is predicted, and the latter evaluates how well our model predicts the shape. The latter metric is defined as:

$$\mathrm{DVO}\left(V_{p},V_{t}\right)=\frac{|V_{p}\cap V_{t}|}{|V_{p}\cup V_{t}|}.$$

**Figure 8:**
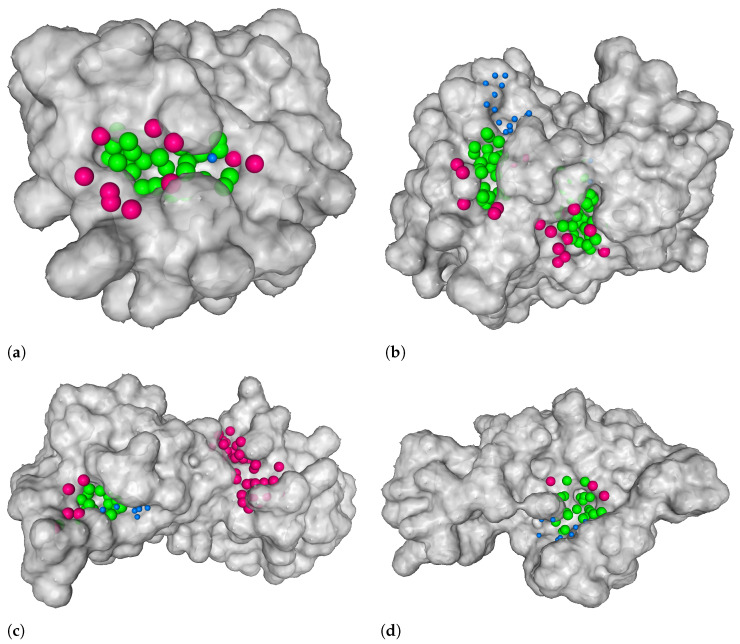
Four examples of our model’s output. The green balls on the protein surface represent points that were correctly predicted as ligandable points (true positives), blue balls represent points that were falsely predicted as non-ligandable points (false negatives), and pink balls represent points that were falsely predicted as ligandable (false positives). True negatives are not shown. In all figures, there are binding sites that were correctly predicted by our model. (**b**) Example where the location of a binding site was not detected—this is depicted with blue balls. (**c**) Example of a predicted binding site that is not present in our dataset—it is depicted with pink balls. (**a**) 4rrg, chain C [41,42]. (**b**) 3lw0, chain D [43,44]. (**c**) 3nk7, chain B [39,40]. (**d**) 1tkg, chain A [45,46].

Note that we adapted the definition of the DVO metric from the one used in DeepSite and DeepPocket. While these original works use the convex hull of a predicted binding site’s points and take the quotient of the number of points in the intersection of convex hulls and their union, we simplify this computation and take the quotient between the intersection of points in the true and predicted binding site and their union. We justify this simplification with two arguments. Firstly, the visual informative value of a convex hull compared to the original point cloud is smaller since it often intersects with the protein. Secondly, the simplified quotient is less sensitive to outliers, i.e., points that make up the binding site but stand out. Such points make a big difference in the shape if you consider the convex hulls but make little difference otherwise. Figure 8 displays four examples of such predicted binding sites. The figure illustrates some good predictions, a missed one, and one of a yet unknown binding site.

**Figure 9:**
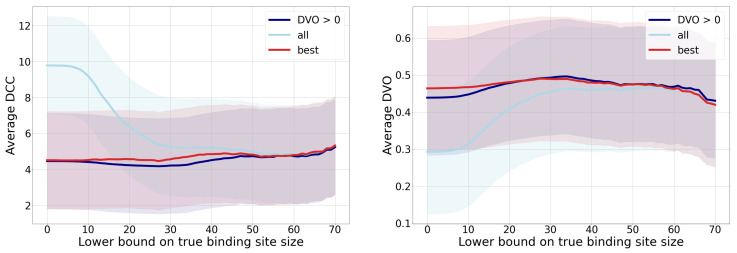
Performance comparison of binding site detection depending on the lower bound on true binding site size. The left graph displays an average distance to the true binding site distance (DCC) (less is better), and the right graph displays the discrete volumetric overlap (DVO) (more is better). Each graph contains three curves for three groups of observed binding sites: for all binding sites, the best in each chain, and for those with DVO>0. The intervals (stripes) around the curves denote the standard deviations of each value—the width of each stripe is two standard deviations. Although the deviations seem large, the absolute values they represent in each metric are expected.

The predictive model’s analysis in terms of the DCC (distance from the binding site center) and the DVO metric is shown in Figure 9 for three different types of binding sites. The first group includes all binding sites, the second group considers only one binding site per each chain with minimum distance (left graph) or maximum DVO (right graph), and the third group is only binding sites with positive a DVO value. Since many binding sites are small, we decided to observe only binding sites that were larger than a pre-chosen true binding site size threshold. We can observe from the figures that the quality of predictions improves by increasing the lower bound of the true binding site size, and that it stabilizes around 30. Considering that pharmacologically relevant ligands are of larger sizes and also require larger binding sites, we can assume that the model’s performance is satisfactory.

Since each chain can contain several binding sites and some of them can be predicted more successfully than others (according to the distance to center and to the DVO), we include in the group “best” only those true binding sites that were the most successfully predicted by our approach. By omitting the less successful binding site targets, the corresponding curve in the graphs expectedly displays an improvement compared to the group of all binding sites. Since fewer successfully predicted binding sites are small, we can observe the biggest difference between both groups in smaller lower bounds of binding site sizes. An additional aspect of the predictive performance is also shown with the curve that denotes all binding sites with DVO>0 (i.e., binding sites that were at least marginally detected). On average, the curve for this group of binding sites is better than the curve for all binding sites for those parts of the graph where there exist multiple chains for which we predicted no binding sites.

Table 3 displays the achieved performance metrics for the described three groups of binding sites and additionally for the group of all binding sites that contain more than 30 points. The table additionally provides a proportion of binding sites for which the predicted center is 4 Å away the most. We can confirm that the provided results also confirm the performance order of groups that was reflected in the graphs in Figure 9.

### 3.2. Classification Performance Metrics

**Figure 10:**
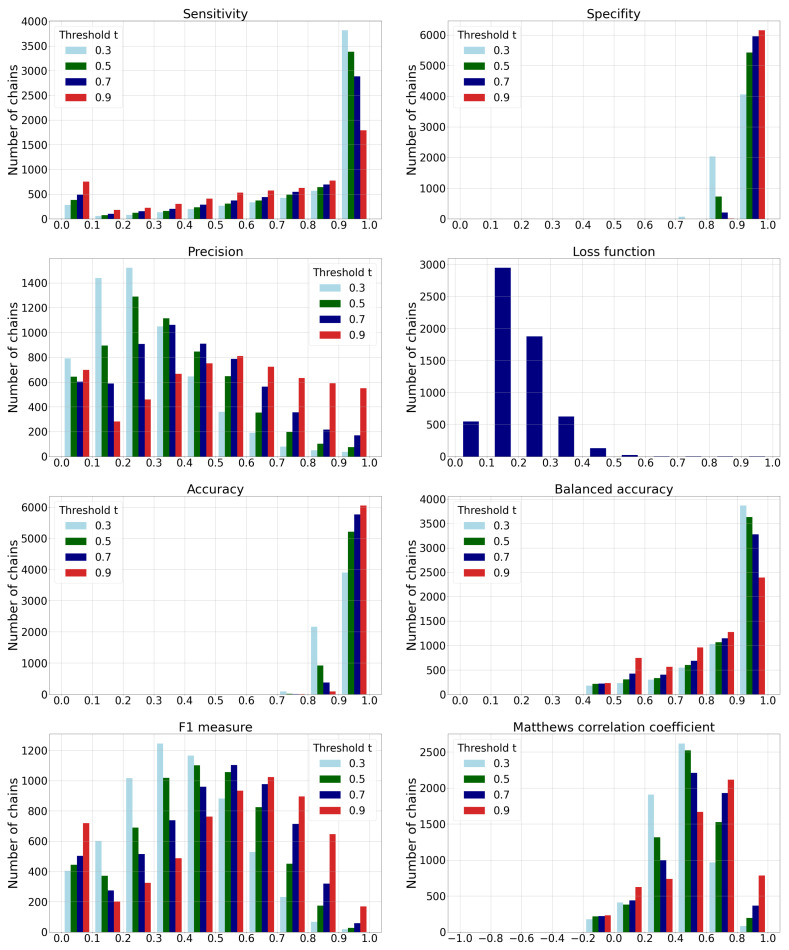
Histograms of different performance metric values. For all metrics except for the loss function, the histograms are shown for different thresholds t∈[0.3,0.5,0.7,0.9]. The data contain joint results obtained on all ten test data sets.

In addition to metrics from the previous subsection, we evaluate the predictive model traditional metrics that are required to justify the further required grouping of the predicted ligandable points into predicted binding sites. If we denote ligandable points as positive examples (*P*) and non-ligandable points as negative examples (*N*), we can compute the traditional machine learning metrics such as: sensitivity, specificity, precision, loss function, accuracy, balanced accuracy, F1 measure, and Matthews correlation coefficient (MCC). Since the protein chains have different numbers of points on the molecular surface, we evaluated the performance for each individual chain and combined the results into a joint histogram. To determine the class of each prediction, we used four different ligandability thresholds (t∈{0.3,0.5,0.7,0.9}) that determine that a point is ligandable when its ligandability prediction is higher than *t*. The results are shown in Figure 10 and Table 4.

## 4. Discussion

We can conclude the following from the presented results:

- The histogram of *sensitivity* and *recall* shows that a smaller-threshold *t* results in more ligandable points. A ratio of predicted true ligandable points is therefore higher with a smaller *t*, while the higher *t* could result in overlooking some of the possible binding sites.
- A higher-threshold *t* on the histogram of *specificity* results in a higher number of non-ligandable points. The ratio of predicted true non-ligandable points (i.e., specificity) is therefore higher with higher values of *t*.
- The *precision* metric reports a ratio of predicted ligandable points that are truly ligandable. A smaller-threshold *t* here indicates worse results; namely, it causes a larger number of false positive points to be predicted. Similarly, a higher *t* causes a smaller number of predicted true ligandable points; hence, the precision remains low.
- The histogram of the loss function is independent of changing the threshold *t* since its value is computed directly from the predicted value for each individual point.
- The histogram of *accuracy* reveals influence of the imbalanced data set. A higher-threshold *t* here causes only a few points to be predicted as ligandable, but as the number of non-ligandable points is high, the resulting accuracy is also high. As the simple solution, we also compute the *balanced accuracy* that is computed as an average of sensitivity and specificity. Their graphs show that the balanced accuracy is low in some parts due to the low sensitivity for some chains. Here, a higher-threshold *t* also causes lower sensitivity.
- The $F_{1}$-score is a harmonic mean between precision and sensitivity. Higher values of $F_{1}$-score mean that both underlying measures are high; while low values can be a result of either one of them being low. If we observe the threshold t=0.3, we can note the high sensitivity and the low precision, resulting in a low $F_{1}$-score. We can also note the best ratio with t=0.9, where the sensitivity is moderate and the precision seems to be the highest.
- Matthews correlation coefficient is a number from the interval [−1,1] that expresses how well model’s predictions are correlated with true values. Values higher than 0 denote positive correlation, and values less than 0 denote negative correlation. A higher value denotes higher predictive capability. We can see from the graph that the best results are achieved with a threshold t=0.9, as the correlation is lower with all the remaining thresholds.

To summarize, the results reveal that one has to be cautious when selecting the threshold *t*, since this impacts the predictive model that detects the ligandable points on different chains with different certainties. This means that the selection of a static threshold *t* would not be an optimal solution for all chains. Namely, predicting as ligandable only points with a prediction higher than *t* would result in especially lowering the resulting sensitivity.

Note that it is not feasible to draw a direct comparison of our model’s performance to the performance of other state-of-the-art methods since each work performed the data acquisition and pre-processing differently. This is especially prominent in our case, because we did not limit ourselves to the selected ligands present in the scPDB and PDBBind databases but acquired all possible ligands for each protein and then merged ligands of similar protein chains, increasing the average number of ligands per protein chain. Nevertheless, we can mention the important results of DeepSite and DeepPocket. DeepSite reports the proportion of predicted binding sites that are closer than 4 Å to the true binding site to be 51%, and DeepPocket reports this value to be between 65% and 80% depending on the dataset at hand. The average value of the DVO metric for DeepSite is 0.652, and at DeepPocket it is between 0.62 and 0.64, again depending on the dataset. We argue one could, with a grain of salt, compare these results to our results of the group “best” or the group of ligands higher than 30 points. Our results are not as good, which was also expected given the more complex data source and different pre-processing phase. Given also that different works used different metrics, this calls for the ambition of proposing standard performance metrics for protein docking methods in the future.

## 5. Conclusions

In the present work, we described the CAT-Site method used to predict binding sites on protein using machine learning. The data were pre-processed using chosen training data, grouping of similar proteins, and pooling of their ligands. An important property of the data set used was knowledge about all the binding sites of proteins, not just some particular ones, which represent the generalization of previous studies. In the next steps, we defined the problem of machine learning using convolutional neural network, and the results were analyzed with metrics typical for the discipline of protein binding site search as well as with metrics commonly used in evaluating the abilities of classification models. We showed that the prediction ability of the method depends strongly on the size of the binding site. On average, the method works better for larger binding sites; however, these are usually less important in pharmacology.

Although the proposed method represents a complete workflow for protein binding site prediction, further work calls for evaluation and validation also from the biochemists’ point of view. It is important to find which chemical properties are relevant for causing part of a protein to become a potential binding site and how these properties interact with each other. Furthermore, most existing binding site prediction methods consist of independent parts that can be considered as interchangeable modules. In the future, it would be interesting to evaluate promising combinations of modules from different research.

The ideas for further work also include computing additional or different atom properties for representation of protein chains and ligands. Further, the use of additional machine learning models could be explored, especially the use of different deep learning neural topologies and ensembles. As mentioned above, the use of proprietary metrics in each work calls for proposing a standard evaluation metric for protein docking methods in the future. Finally, since our method uses several parameters and thresholds (e.g., the upper bound for similar enough chains from Equation (2), the parameters for solvent-accessible surface point computation, and the choice of percentiles when combining ligandability predictions), methods for automatic setting of the optimal parameters shall be explored.

Even though further testing and evaluation of the proposed method are needed, CAT-Site represents a promising comprehensive approach to solve a complex problem of ligand binding to proteins—a problem that is important not only in pharmaceutical industry but also for studying biological processes. While molecular docking studies usually focus on predicting the ligand poses and the binding constants on the protein, knowing the possible binding sites on the protein that can be identified through CAT-Site is important to predict the efficiency of newly synthesized drugs as well as their potential side-effects. Further, these binding sites might potentially lead to protein–protein association, which causes destabilization of biological solutions. Further work, however, is needed to test this potential applicability.

## Figures and Tables

**Table 1:** Chosen types of atoms according to their chemical properties.

Atom Type	Description
C	Non H-bonding aliphatic carbon
A	Non H-bonding aromatic carbon
NA	Nitrogen, one H-bond acceptor
NS	Nitrogen acceptor of NS type
OA	Oxygen, two H-bonds acceptor
OS	Oxygen acceptor of type OS
SA	Sulfur, two H-bonds acceptor
HD	Hydrogen, donor of one H-bond
HS	Hydrogen, donor of HS type
MG	Magnesium, not binding to hydrogen
ZN	Zinc, not binding to hydrogen
MN	Manganese, not binding to hydrogen
CA	Calcium, not binding to hydrogen
FE	Iron, not binding to hydrogen

**Table 2:** Chosen types of atoms arranged into eight channels.

Property	Examples
hydrophobicity	atoms of type C or A
aromaticity	atoms of type A
H-bond acceptors	atoms of type NA, or NS, or OA, or OS, or SA
H-bond donor	atoms of type HD, or HS partnering with O or N
positive ionizability	positively charged atoms
negative ionizability	negatively charged atoms
metal	atoms of type MG, or ZN, or MN, or CA, or FE
excluded volume	all atom types (including the ones not given in Table 1)

**Table 3:** The achieved values of the distances to binding site center and discrete volumetric overlap (DVO) for four groups of binding sites. The table cells display values in the form average±standarddeviation, obtained from 10 models that were evaluated on independent test sets.

Metric\Group	All	All > 30	Best	DVO > 0
distance	9.780±0.614	5.284±0.462	4.519±0.299	3.536±0.128
DVO	0.293±0.013	0.455±0.012	0.464±0.010	0.508±0.007
distance≤4 Å	0.378±0.030	0.540±0.032	0.634±0.025	0.668±0.023

**Table 4:** Values of the observed performance metrics for different thresholds *t*. Values in table cells are in the form average±standarddeviation and are computed on 10 models obtained from independent test sets. The best values for each metric are denoted with bold.

Threshold *t*	0.3	0.5	0.7	0.9
sensitivity	0.829±0.010	0.789±0.011	0.743±0.013	0.635±0.019
specificity	0.916±0.004	0.942±0.003	0.960±0.003	0.980±0.002
precision	0.282±0.009	0.346±0.010	0.408±0.014	0.511±0.019
loss function	0.201±0.010	0.201±0.010	0.201±0.010	0.201±0.010
accuracy	0.911±0.004	0.934±0.003	0.950±0.003	0.965±0.002
balanced acc.	0.872±0.005	0.865±0.006	0.851±0.006	0.807±0.009
$F_{1}$-score	0.387±0.010	0.441±0.012	0.481±0.014	0.517±0.015
MCC	0.430±0.010	0.473±0.011	0.504±0.013	0.529±0.014

## Data Availability

Data is contained within the article.

## Abbreviations

The following abbreviations are used in this manuscript:

CNN	Convolutional Neural Network
SAS	Solvent Accessible Surface
DVO	Discrete Volumetric Overlap
DCC	Distance from Center to Center
MCC	Matthews Correlation Coefficient
PDB	Protein Data Bank
